# 9-Chloro-2,4-dimethoxy­acridinium trifluoro­methane­sulfonate

**DOI:** 10.1107/S1600536809008551

**Published:** 2009-03-14

**Authors:** Beata Zadykowicz, Karol Krzymiński, Damian Trzybiński, Artur Sikorski, Jerzy Błażejowski

**Affiliations:** a Faculty of Chemistry, University of Gdańsk, J. Sobieskiego 18, 80-952 Gdańsk, Poland

## Abstract

In the mol­ecular structure of the title compound, C_15_H_13_ClNO_2_^+^·CF_3_SO_3_^−^, the meth­oxy groups are nearly coplanar with the acridine ring system, making dihedral angles of 0.4 (2) and 5.1 (2)°. Multidirectional π–π contacts between acridine units are observed in the crystal structure. N—H⋯O and C—H⋯O hydrogen bonds link cations and anions, forming a layer structure.

## Related literature

For general background, see: Acheson (1973 ▶); Demeunynck *et al.* (2001 ▶); Wróblewska *et al.* (2004 ▶); Zomer & Jacquemijns (2001 ▶). For related structures, see: Achari & Neidle (1977 ▶); Neidle (1982 ▶); Ning *et al.* (1976 ▶); Ojida *et al.* (2006 ▶); Rimmer *et al.* (2000 ▶); Toma *et al.* (1993 ▶). For inter­molecular inter­actions, see: Aakeröy *et al.* (1992 ▶); Bianchi *et al.* (2004 ▶); Hunter *et al.* (2001 ▶); Steiner (1999 ▶). For the synthesis, see: Acheson (1973 ▶); Sato (1996 ▶). For a description of the Cambridge Structural Database, see: Allen (2002 ▶).
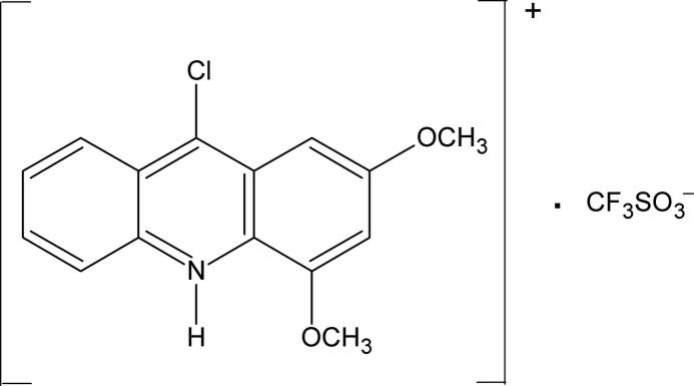


## Experimental

### 

#### Crystal data

C_15_H_13_ClNO_2_^+^·CF_3_SO_3_^−^
*M* *_r_* = 423.79
Monoclinic, 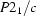

*a* = 11.0502 (9) Å
*b* = 23.110 (2) Å
*c* = 7.1435 (8) Å
β = 108.214 (11)°
*V* = 1732.8 (3) Å^3^
*Z* = 4
Mo *K*α radiation
μ = 0.40 mm^−1^
*T* = 295 K
0.60 × 0.20 × 0.10 mm

#### Data collection

Oxford Diffraction Gemini R Ultra Ruby CCD diffractometer
Absorption correction: multi-scan (*CrysAlis RED*; Oxford Diffraction, 2008 ▶) *T*_min_ = 0.782, *T*_max_ = 0.959
12548 measured reflections
3072 independent reflections
1932 reflections with *I* > 2σ(*I*)
*R*_int_ = 0.063

#### Refinement

*R*[*F* ^2^ > 2σ(*F* ^2^)] = 0.058
*wR*(*F* ^2^) = 0.179
*S* = 1.06
3072 reflections
246 parameters
H-atom parameters constrained
Δρ_max_ = 0.41 e Å^−3^
Δρ_min_ = −0.28 e Å^−3^

### 

Data collection: *CrysAlis CCD* (Oxford Diffraction, 2008 ▶); cell refinement: *CrysAlis RED* (Oxford Diffraction, 2008 ▶); data reduction: *CrysAlis RED*; program(s) used to solve structure: *SHELXS97* (Sheldrick, 2008 ▶); program(s) used to refine structure: *SHELXL97* (Sheldrick, 2008 ▶); molecular graphics: *ORTEP-3* (Farrugia, 1997 ▶); software used to prepare material for publication: *SHELXL97* and *PLATON* (Spek, 2009 ▶).

## Supplementary Material

Crystal structure: contains datablocks global, I. DOI: 10.1107/S1600536809008551/xu2477sup1.cif

Structure factors: contains datablocks I. DOI: 10.1107/S1600536809008551/xu2477Isup2.hkl

Additional supplementary materials:  crystallographic information; 3D view; checkCIF report

## Figures and Tables

**Table 1:** Hydrogen-bond geometry (Å, °)

*D*—H⋯*A*	*D*—H	H⋯*A*	*D*⋯*A*	*D*—H⋯*A*
N10—H10⋯O23	0.86	2.01	2.826 (4)	159
C5—H5⋯O23	0.93	2.42	3.151 (5)	136
C8—H8⋯O22^i^	0.93	2.53	3.348 (6)	147
C18—H18*A*⋯O22	0.96	2.56	3.326 (5)	137
C18—H18*C*⋯O22^ii^	0.96	2.55	3.462 (5)	158

Symmetry codes: (i) 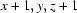; (ii) 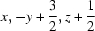.

**Table 2:** π–π Interactions (Å,°)

*CgI*	*CgJ*	*Cg*⋯*Cg*	Dihedral angle	Interplanar distance
1	1^iii^	3.817 (2)	0.33	3.506 (2)
1	1^ii^	3.817 (2)	0.33	3.499 (2)
1	2^iii^	3.984 (2)	1.19	3.484 (2)
1	2^ii^	3.616 (2)	1.19	3.493 (2)
2	3^iii^	3.919 (2)	1.95	3.490 (2)
3	2^ii^	3.919 (2)	1.95	3.509 (2)

Symmetry codes: (ii) 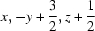; (iii) 
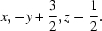
 *Cg*1, *Cg*2 and *Cg*3 are the centroids of the C9/N10/C11–C14, C1–C4/C11/C12 and C5–C8/C13/C14 rings, respectively. *Cg*⋯*Cg* is the distance between ring centroids. The dihedral angle is that between the planes of the rings. The interplanar distance is the perpendicular distance of *CgI* from ring *J*.

**Fig. 1.**
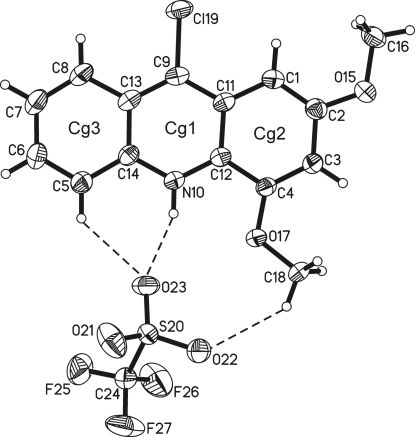
The molecular structure of the title compound showing the atom-labeling scheme. Displacement ellipsoids are drawn at the 25% probability level and H atoms are shown as small spheres of arbitrary radius. The C5–H5···O23, N10–H10···O23 and C18–H18A···O22 hydrogen bonds are represented by dashed lines. Cg1, Cg2 and Cg3 denote the ring centroids.

**Fig. 2.**
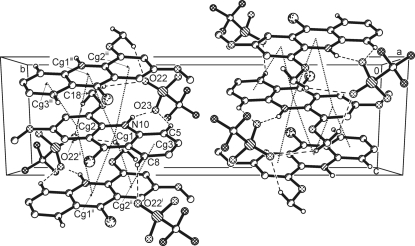
The arrangement of the ions in the crystal structure, viewed along the a axis. The N–H···O and C–H···O hydrogen bonds are represented by dashed lines, the π-π contacts by dotted lines. H atoms not involved in interactions have been omitted. [Symmetry codes: (i) x + 1, y, z + 1; (ii) x, -y + 3/2, z + 1/2; (iii) x, -y + 3/2, z - 1/2.]

**Fig. 3.**
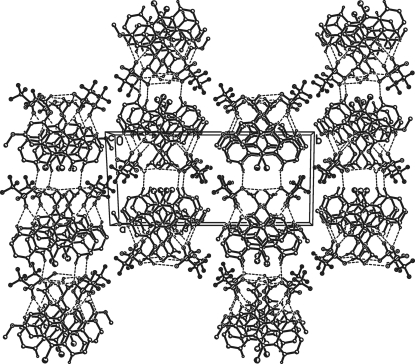
Ion pair stacks in the crystal structure, viewed along the c axis. The N–H···O and C–H···O interactions are represented by dashed lines. H atoms not involved in interactions have been omitted.

## supplementary crystallographic information

### Comment

Acridinium cations containing various substituents at position 9 and alkyl-substituted at the endocyclic N atom (position 10) undergo oxidation by H_2_O_2_ or other peroxides in alkaline media, which leads to the formation of electronically excited 10-alkyl-9-acridinones capable of emitting light with a quantum yield of several percent (Zomer & Jacquemijns, 2001; Wróblewska *et al.*, 2004). This chemiluminescence is affected by the features of the substituent at position 9 and by the constitution of the acridine fragment. In the search for derivatives that could exhibit an enhanced chemiluminogenic ability we turned our attention to compounds in which the C atom at position 9 is bound to a Cl atom. One of the compounds synthesized was 9-chloro-2,4-dimethoxyacridinium trifluoromethanesulfonate. This compound was obtained by the reaction of 9-chloro-2,4-dimethoxyacridine with methyl trifluoromethanesulfonate, which usually leads to quarternarization of the endocyclic N atom (Sato, 1996). Since this did not happen, it is possible that traces of water caused the transformation of methyl trifluoromethanesulfonate to trifluoromethanesulfonic acid and methanol, and the reaction of the former entity with 9-chloro-2,4-dimethoxyacridine to yield 9-chloro-2,4-dimethoxyacridinium trifluoromethanesulfonate. The cation of the title compound has a protonated endocyclic N-atom, which makes its reaction with oxidants possible. On the other hand, substitution in the acridine moiety by two methoxy groups should cause a red shift of the chemiluminescence emission, advantageous in analytical applications. 9-Chloroacridines have been precursors of numerous 9-substitued acridine derivatives (Acheson, 1973; Wróblewska *et al.*, 2004), including drugs (Acheson, 1973; Demeunynck *et al.*, 2001). This paper presents the crystal structure of the title compound.

The acridine units, with an average deviation from planarity of 0.025 (5) Å, are parallel in the crystal lattice. In the cation of the title compound (Fig. 1) the bond lengths and angles characterizing the geometry of the 9-chloroacridine skeleton are similar to those in 9-chloroacridine itself (Achari & Neidle, 1977), a 9-chloroacridine derivative (Neidle, 1982), a 9-chloroacridine iodine complex (Rimmer *et al.*, 2000), two solvates of 9-chloroacridine derivatives (Toma *et al.*, 1993; Ojida *et al.*, 2006) and the salt-type compound containing the 9-chloroacridinium cation (Ning *et al.*, 1976). The crystal structures of these six compounds were found in the Cambridge Structural Database (Version 5.29; Allen, 2002). The C(9)–Cl, N(10)–C(12) and N(10)–C(14) bond lengths (in Å) in them vary from 1.719 to 1.748, from 1.332 to 1.375 and from 1.349 to 1.383, respectively. The corresponding values for the compound investigated (1.723, 1.346 and 1.351) thus fall well within the ranges found for other 9-chloroacridines.

In the crystal structure, N–H···O (Aakeröy *et. al*., 1992) and C–H···O (Steiner, 1999; Bianchi *et al.*, 2004) hydrogen bonds link cations and anions in ion pairs (Table 1, Fig. 1). Inversely oriented ion pairs form stacks *via*π-π contacts of an attractive nature (Hunter *et al.*, 2001), involving the central ring (*Cg*1) and the aromatic rings (*Cg*2 and *Cg*3) (Table 2), as well as C–H···O interactions between adjacent ions (Fig. 2). Stacks arranged in parallel are linked through intermolecular C–H···O interactions (Figs 2 and 3) to form layers (Fig. 3). The crystal structure is stabilized by short-range non-specific dispersive interactions between inversely oriented layers (Fig. 3) as well as by long-range electrostatic interactions between ions.

### Experimental

9-Chloro-2,4-dimethoxyacridine was prepared by heating 2-[(2,4-dimethoxyphenyl)amino]benzoic acid, obtained as described elsewhere (Acheson, 1973), with a sevenfold molar excess of POCl_3_ (400 K, 3 h). The excess POCl_3_ was subsequently removed under reduced pressure. The residue was dispersed in CHCl_3_, stirred in the presence of a mixture of ice and aqueous ammonia, separated by filtration and dried. The crude product was purified chromatographically (neutral Al_2_O_3_, CHCl_3_/toluene, 1/1 *v*/*v*, *R*_f_=0.29). The 9-chloro-2,4-dimethoxyacridine was dissolved in CH_2_Cl_2_, then treated with a fivefold molal excess of methyl trifluoromethanesulfonate dissolved in the same solvent (under an Ar atmosphere at room temperature for 3 h) (Sato, 1996). The crude salt was dissolved in a small amount of ethanol, filtered and precipitated with a 25 *v*/*v* excess of diethyl ether (yield: 71%). Purple crystals suitable for X-ray investigations were grown from 2-propanol solution (m.p. 493–496 K).

### Refinement

H atoms were positioned geometrically, with C—H = 0.93 and 0.96 Å for the aromatic and methyl H atoms, respectively, and constrained to ride on their parent atoms with *U*_iso_(H) = *xU*_eq_(C), where *x* = 1.2 for the aromatic and *x* = 1.5 for the methyl H atoms.

### Crystal data

**Table:**

C_15_H_13_ClNO_2_^+^·CF_3_SO_3_^−^	*F*(000) = 864
*M**_r_* = 423.79	*D*_x_ = 1.625 Mg m^−^^3^
Monoclinic, *P*2_1_/*c*	Mo *K*α radiation, λ = 0.71073 Å
Hall symbol: -P 2ybc	Cell parameters from 5776 reflections
*a* = 11.0502 (9) Å	θ = 3.0–29.2°
*b* = 23.110 (2) Å	µ = 0.40 mm^−^^1^
*c* = 7.1435 (8) Å	*T* = 295 K
β = 108.214 (11)°	Plate, purple
*V* = 1732.8 (3) Å^3^	0.60 × 0.20 × 0.10 mm
*Z* = 4	

### Data collection

**Table:**

Oxford Diffraction Gemini R Ultra Ruby CCD diffractometer	3072 independent reflections
Radiation source: Enhance (Mo) X-ray Source	1932 reflections with *I* > 2σ(*I*)
graphite	*R*_int_ = 0.063
Detector resolution: 10.4002 pixels mm^-1^	θ_max_ = 25.1°, θ_min_ = 3.1°
ω scans	*h* = −13→13
Absorption correction: multi-scan (*CrysAlis RED*; Oxford Diffraction, 2008)	*k* = −25→27
*T*_min_ = 0.782, *T*_max_ = 0.959	*l* = −8→8
12548 measured reflections	

### Refinement

**Table:**

Refinement on *F*^2^	Primary atom site location: structure-invariant direct methods
Least-squares matrix: full	Secondary atom site location: difference Fourier map
*R*[*F*^2^ > 2σ(*F*^2^)] = 0.058	Hydrogen site location: inferred from neighbouring sites
*wR*(*F*^2^) = 0.179	H-atom parameters constrained
*S* = 1.06	*w* = 1/[σ^2^(*F*_o_^2^) + (0.1062*P*)^2^*P*] where *P* = (*F*_o_^2^ + 2*F*_c_^2^)/3
3072 reflections	(Δ/σ)_max_ < 0.001
246 parameters	Δρ_max_ = 0.41 e Å^−^^3^
0 restraints	Δρ_min_ = −0.28 e Å^−^^3^

### Special details

**Table:**

Geometry. All e.s.d.'s (except the e.s.d. in the dihedral angle between two l.s. planes) are estimated using the full covariance matrix. The cell e.s.d.'s are taken into account individually in the estimation of e.s.d.'s in distances, angles and torsion angles; correlations between e.s.d.'s in cell parameters are only used when they are defined by crystal symmetry. An approximate (isotropic) treatment of cell e.s.d.'s is used for estimating e.s.d.'s involving l.s. planes.

### Fractional atomic coordinates and isotropic or equivalent isotropic displacement parameters (Å^2^)

**Table:**

	*x*	*y*	*z*	*U*_iso_*/*U*_eq_	
C1	1.1372 (3)	0.84277 (15)	0.6837 (5)	0.0588 (9)	
H1	1.2152	0.8604	0.7447	0.071*	
C2	1.0316 (3)	0.87560 (16)	0.6029 (5)	0.0630 (9)	
C3	0.9120 (3)	0.85005 (15)	0.5039 (5)	0.0606 (9)	
H3	0.8414	0.8734	0.4478	0.073*	
C4	0.9004 (3)	0.79165 (14)	0.4907 (5)	0.0539 (8)	
C5	1.0717 (4)	0.60054 (16)	0.6391 (5)	0.0672 (9)	
H5	0.9905	0.5857	0.5802	0.081*	
C6	1.1712 (4)	0.56449 (18)	0.7170 (6)	0.0774 (11)	
H6	1.1578	0.5247	0.7103	0.093*	
C7	1.2939 (4)	0.5859 (2)	0.8075 (6)	0.0824 (12)	
H7	1.3608	0.5603	0.8595	0.099*	
C8	1.3169 (4)	0.64432 (19)	0.8204 (6)	0.0740 (10)	
H8	1.3990	0.6579	0.8812	0.089*	
C9	1.2292 (3)	0.74378 (17)	0.7518 (5)	0.0586 (9)	
N10	0.9955 (2)	0.69822 (11)	0.5708 (4)	0.0525 (7)	
H10	0.9213	0.6840	0.5128	0.063*	
C11	1.1281 (3)	0.78210 (15)	0.6748 (4)	0.0532 (8)	
C12	1.0075 (3)	0.75623 (14)	0.5787 (4)	0.0507 (8)	
C13	1.2163 (3)	0.68400 (16)	0.7418 (5)	0.0606 (9)	
C14	1.0924 (3)	0.66091 (15)	0.6481 (4)	0.0552 (8)	
O15	1.0275 (2)	0.93401 (11)	0.6023 (4)	0.0789 (8)	
C16	1.1437 (4)	0.96502 (17)	0.6957 (6)	0.0822 (11)	
H16A	1.1261	1.0057	0.6933	0.123*	
H16B	1.2038	0.9577	0.6263	0.123*	
H16C	1.1787	0.9523	0.8298	0.123*	
O17	0.7946 (2)	0.76076 (10)	0.3978 (4)	0.0661 (7)	
C18	0.6815 (3)	0.79154 (16)	0.2905 (6)	0.0708 (10)	
H18A	0.6146	0.7644	0.2313	0.106*	
H18B	0.6985	0.8145	0.1894	0.106*	
H18C	0.6557	0.8164	0.3789	0.106*	
Cl19	1.37790 (8)	0.77318 (5)	0.86037 (16)	0.0835 (4)	
S20	0.67506 (9)	0.60826 (4)	0.27655 (15)	0.0686 (4)	
O21	0.7046 (4)	0.56795 (15)	0.1495 (6)	0.1301 (14)	
O22	0.5948 (3)	0.65462 (13)	0.1825 (5)	0.1074 (11)	
O23	0.7793 (2)	0.62580 (14)	0.4408 (5)	0.0967 (9)	
C24	0.5785 (4)	0.56702 (17)	0.3904 (6)	0.0738 (10)	
F25	0.6355 (3)	0.52276 (13)	0.4837 (5)	0.1314 (12)	
F26	0.5357 (4)	0.59810 (14)	0.5057 (6)	0.1486 (14)	
F27	0.4759 (3)	0.54695 (15)	0.2592 (5)	0.1493 (14)	

### Atomic displacement parameters (Å^2^)

**Table:**

	*U*^11^	*U*^22^	*U*^33^	*U*^12^	*U*^13^	*U*^23^
C1	0.054 (2)	0.065 (2)	0.0553 (19)	−0.0088 (16)	0.0140 (17)	−0.0015 (16)
C2	0.063 (2)	0.063 (2)	0.065 (2)	−0.0052 (18)	0.0233 (19)	0.0038 (17)
C3	0.052 (2)	0.060 (2)	0.069 (2)	0.0021 (16)	0.0174 (17)	0.0038 (16)
C4	0.0440 (19)	0.060 (2)	0.0566 (19)	0.0002 (15)	0.0145 (16)	0.0040 (15)
C5	0.063 (2)	0.068 (2)	0.066 (2)	0.0039 (18)	0.0139 (18)	0.0018 (18)
C6	0.085 (3)	0.068 (2)	0.075 (2)	0.021 (2)	0.019 (2)	0.004 (2)
C7	0.069 (3)	0.091 (3)	0.078 (3)	0.027 (2)	0.010 (2)	0.001 (2)
C8	0.054 (2)	0.092 (3)	0.068 (2)	0.016 (2)	0.0068 (18)	0.002 (2)
C9	0.0459 (19)	0.079 (2)	0.0499 (19)	−0.0051 (17)	0.0143 (16)	−0.0011 (16)
N10	0.0432 (15)	0.0611 (17)	0.0512 (15)	−0.0001 (12)	0.0117 (12)	0.0015 (12)
C11	0.0461 (19)	0.070 (2)	0.0451 (17)	0.0013 (16)	0.0166 (15)	0.0014 (15)
C12	0.048 (2)	0.061 (2)	0.0460 (17)	0.0001 (15)	0.0194 (15)	−0.0001 (14)
C13	0.047 (2)	0.083 (3)	0.0505 (19)	0.0091 (17)	0.0132 (16)	0.0031 (17)
C14	0.052 (2)	0.065 (2)	0.0497 (18)	0.0068 (16)	0.0174 (16)	0.0027 (16)
O15	0.0707 (17)	0.0567 (15)	0.1022 (19)	−0.0073 (13)	0.0168 (15)	−0.0006 (13)
C16	0.082 (3)	0.067 (2)	0.087 (3)	−0.022 (2)	0.011 (2)	−0.002 (2)
O17	0.0444 (14)	0.0601 (14)	0.0823 (17)	−0.0032 (11)	0.0030 (12)	0.0067 (12)
C18	0.045 (2)	0.071 (2)	0.086 (3)	0.0088 (17)	0.0050 (19)	0.0042 (19)
Cl19	0.0457 (6)	0.1037 (8)	0.0908 (7)	−0.0074 (5)	0.0067 (5)	−0.0076 (6)
S20	0.0630 (6)	0.0553 (6)	0.0846 (7)	−0.0041 (4)	0.0190 (5)	0.0067 (5)
O21	0.186 (4)	0.090 (2)	0.156 (3)	−0.022 (2)	0.114 (3)	−0.022 (2)
O22	0.082 (2)	0.0794 (19)	0.138 (3)	−0.0040 (16)	0.0005 (19)	0.0429 (18)
O23	0.0595 (16)	0.099 (2)	0.115 (2)	−0.0209 (16)	0.0038 (16)	0.0111 (18)
C24	0.063 (2)	0.066 (2)	0.089 (3)	−0.001 (2)	0.021 (2)	0.005 (2)
F25	0.112 (2)	0.109 (2)	0.178 (3)	0.0179 (17)	0.052 (2)	0.080 (2)
F26	0.165 (3)	0.122 (2)	0.210 (4)	−0.025 (2)	0.132 (3)	−0.023 (2)
F27	0.111 (2)	0.174 (3)	0.136 (3)	−0.081 (2)	−0.001 (2)	0.033 (2)

### Geometric parameters (Å, °)

**Table:**

C1—C2	1.360 (5)	N10—C12	1.346 (4)
C1—C11	1.405 (5)	N10—C14	1.351 (4)
C1—H1	0.9300	N10—H10	0.8600
C2—O15	1.351 (4)	C11—C12	1.426 (4)
C2—C3	1.418 (5)	C13—C14	1.427 (5)
C3—C4	1.356 (5)	O15—C16	1.439 (4)
C3—H3	0.9300	C16—H16A	0.9600
C4—O17	1.354 (4)	C16—H16B	0.9600
C4—C12	1.414 (4)	C16—H16C	0.9600
C5—C6	1.353 (5)	O17—C18	1.434 (4)
C5—C14	1.412 (5)	C18—H18A	0.9600
C5—H5	0.9300	C18—H18B	0.9600
C6—C7	1.398 (6)	C18—H18C	0.9600
C6—H6	0.9300	S20—O21	1.408 (3)
C7—C8	1.370 (6)	S20—O22	1.419 (3)
C7—H7	0.9300	S20—O23	1.422 (3)
C8—C13	1.415 (5)	S20—C24	1.803 (4)
C8—H8	0.9300	C24—F25	1.275 (4)
C9—C13	1.388 (5)	C24—F26	1.289 (4)
C9—C11	1.396 (5)	C24—F27	1.310 (5)
C9—Cl19	1.723 (3)		
			
C2—C1—C11	119.9 (3)	N10—C12—C11	120.2 (3)
C2—C1—H1	120.1	C4—C12—C11	119.8 (3)
C11—C1—H1	120.1	C9—C13—C8	124.7 (3)
O15—C2—C1	125.6 (3)	C9—C13—C14	117.7 (3)
O15—C2—C3	112.9 (3)	C8—C13—C14	117.6 (4)
C1—C2—C3	121.4 (3)	N10—C14—C5	121.1 (3)
C4—C3—C2	120.1 (3)	N10—C14—C13	118.3 (3)
C4—C3—H3	119.9	C5—C14—C13	120.6 (3)
C2—C3—H3	119.9	C2—O15—C16	118.2 (3)
O17—C4—C3	127.4 (3)	O15—C16—H16A	109.5
O17—C4—C12	112.8 (3)	O15—C16—H16B	109.5
C3—C4—C12	119.9 (3)	H16A—C16—H16B	109.5
C6—C5—C14	119.4 (4)	O15—C16—H16C	109.5
C6—C5—H5	120.3	H16A—C16—H16C	109.5
C14—C5—H5	120.3	H16B—C16—H16C	109.5
C5—C6—C7	121.2 (4)	C4—O17—C18	118.4 (3)
C5—C6—H6	119.4	O17—C18—H18A	109.5
C7—C6—H6	119.4	O17—C18—H18B	109.5
C8—C7—C6	120.9 (4)	H18A—C18—H18B	109.5
C8—C7—H7	119.6	O17—C18—H18C	109.5
C6—C7—H7	119.6	H18A—C18—H18C	109.5
C7—C8—C13	120.4 (4)	H18B—C18—H18C	109.5
C7—C8—H8	119.8	O21—S20—O22	115.5 (2)
C13—C8—H8	119.8	O21—S20—O23	115.4 (2)
C13—C9—C11	123.7 (3)	O22—S20—O23	113.59 (19)
C13—C9—Cl19	118.9 (3)	O21—S20—C24	103.3 (2)
C11—C9—Cl19	117.4 (3)	O22—S20—C24	104.03 (19)
C12—N10—C14	124.3 (3)	O23—S20—C24	102.66 (19)
C12—N10—H10	117.9	F25—C24—F26	109.4 (4)
C14—N10—H10	117.9	F25—C24—F27	105.4 (4)
C9—C11—C1	125.3 (3)	F26—C24—F27	104.2 (4)
C9—C11—C12	115.8 (3)	F25—C24—S20	113.3 (3)
C1—C11—C12	118.8 (3)	F26—C24—S20	112.4 (3)
N10—C12—C4	120.0 (3)	F27—C24—S20	111.5 (3)
			
C11—C1—C2—O15	179.7 (3)	Cl19—C9—C13—C8	2.0 (5)
C11—C1—C2—C3	−1.8 (5)	C11—C9—C13—C14	−0.3 (5)
O15—C2—C3—C4	179.7 (3)	Cl19—C9—C13—C14	−179.0 (2)
C1—C2—C3—C4	1.1 (5)	C7—C8—C13—C9	178.3 (3)
C2—C3—C4—O17	−178.2 (3)	C7—C8—C13—C14	−0.7 (5)
C2—C3—C4—C12	1.0 (5)	C12—N10—C14—C5	178.1 (3)
C14—C5—C6—C7	0.5 (6)	C12—N10—C14—C13	−1.1 (4)
C5—C6—C7—C8	0.2 (6)	C6—C5—C14—N10	179.6 (3)
C6—C7—C8—C13	−0.1 (6)	C6—C5—C14—C13	−1.2 (5)
C13—C9—C11—C1	179.4 (3)	C9—C13—C14—N10	1.5 (4)
Cl19—C9—C11—C1	−2.0 (4)	C8—C13—C14—N10	−179.5 (3)
C13—C9—C11—C12	−1.2 (4)	C9—C13—C14—C5	−177.7 (3)
Cl19—C9—C11—C12	177.4 (2)	C8—C13—C14—C5	1.3 (5)
C2—C1—C11—C9	180.0 (3)	C1—C2—O15—C16	−0.5 (5)
C2—C1—C11—C12	0.6 (5)	C3—C2—O15—C16	−179.0 (3)
C14—N10—C12—C4	179.1 (3)	C3—C4—O17—C18	3.6 (5)
C14—N10—C12—C11	−0.6 (4)	C12—C4—O17—C18	−175.7 (3)
O17—C4—C12—N10	−2.6 (4)	O21—S20—C24—F25	59.0 (4)
C3—C4—C12—N10	178.1 (3)	O22—S20—C24—F25	−180.0 (3)
O17—C4—C12—C11	177.1 (3)	O23—S20—C24—F25	−61.4 (4)
C3—C4—C12—C11	−2.2 (4)	O21—S20—C24—F26	−176.3 (4)
C9—C11—C12—N10	1.7 (4)	O22—S20—C24—F26	−55.3 (4)
C1—C11—C12—N10	−178.9 (3)	O23—S20—C24—F26	63.3 (4)
C9—C11—C12—C4	−178.0 (3)	O21—S20—C24—F27	−59.7 (4)
C1—C11—C12—C4	1.4 (4)	O22—S20—C24—F27	61.3 (4)
C11—C9—C13—C8	−179.3 (3)	O23—S20—C24—F27	179.9 (3)

### Hydrogen-bond geometry (Å, °)

**Table:**

*D*—H···*A*	*D*—H	H···*A*	*D*···*A*	*D*—H···*A*
N10—H10···O23	0.86	2.01	2.826 (4)	159
C5—H5···O23	0.93	2.42	3.151 (5)	136
C8—H8···O22^i^	0.93	2.53	3.348 (6)	147
C18—H18A···O22	0.96	2.56	3.326 (5)	137
C18—H18C···O22^ii^	0.96	2.55	3.462 (5)	158

Symmetry codes: (i) *x*+1, *y*, *z*+1; (ii) *x*, −*y*+3/2, *z*+1/2.

### Table 2 π–π Interactions (Å,°).

**Table:**

*CgI*	*CgJ*	*Cg*···*Cg*	Dihedral angle	Interplanar distance
1	1^iii^	3.817 (2)	0.33	3.506 (2)
1	1^ii^	3.817 (2)	0.33	3.499 (2)
1	2^iii^	3.984 (2)	1.19	3.484 (2)
1	2^ii^	3.616 (2)	1.19	3.493 (2)
2	3^iii^	3.919 (2)	1.95	3.490 (2)
3	2^ii^	3.919 (2)	1.95	3.509 (2)

Symmetry codes: (ii) x, -y+3/2, z+1/2; (iii) x, -y+3/2, z-1/2. *Cg*1, *Cg*2 and *Cg*3 are the centroids of the C9/N10/C11–C14, C1–C4/C11/C12 and C5–C8/C13/C14 rings, respectively. Cg···Cg is the distance between ring centroids. The dihedral angle is that between the planes of the rings. The interplanar distance is the perpendicular distance of *CgI* from ring *J*.
